# Association between regular physical exercise and depressive symptoms mediated through social support and resilience in Japanese company workers: a cross-sectional study

**DOI:** 10.1186/s12889-016-3251-2

**Published:** 2016-07-12

**Authors:** Eisho Yoshikawa, Daisuke Nishi, Yutaka J. Matsuoka

**Affiliations:** Department of Neuropsychiatry, Nippon Medical School Tama Nagayama Hospital, 1-7-1 Nagayama Tama City, Tokyo, 206-8512 Japan; Department of Neuropsychiatry, Nippon Medical School, 1-1-5 Sendagi, Bunkyo, Tokyo, 113-8602 Japan; Department of Psychiatry, National Disaster Medical Center, 3256 Midoricho, Tachikawa, Tokyo, 190-0014 Japan; Department of Mental Health Policy and Evaluation, National Institute of Mental Health, National Center of Neurology and Psychiatry, 4-1-1 Ogawahigashi-cho, Kodaira, Tokyo, 187-8553 Japan; Division of Health Care Research, Center for Public Health Sciences, National Cancer Center, 5-1-1 Tsukiji, Chuo-ku, Tokyo, 104-0045 Japan

**Keywords:** Depressive symptoms, Social support, Resilience, Physical exercise

## Abstract

**Background:**

Regular physical exercise has been reported to reduce depressive symptoms. Several lines of evidence suggest that physical exercise may prevent depression by promoting social support or resilience, which is the ability to adapt to challenging life conditions. The aim of this study was to compare depressive symptoms, social support, and resilience between Japanese company workers who engaged in regular physical exercise and workers who did not exercise regularly. We also investigated whether regular physical exercise has an indirect association with depressive symptoms through social support and resilience.

**Methods:**

Participants were 715 Japanese employees at six worksites. Depressive symptoms were assessed with the Center for Epidemiologic Studies Depression (CES-D) scale, social support with the short version of the Social Support Questionnaire (SSQ), and resilience with the 14-item Resilience Scale (RS-14). A self-report questionnaire, which was extracted from the Japanese version of the Health-Promoting Lifestyle Profile, was used to assess whether participants engage in regular physical exercise, defined as more than 20 min, three or more times per week. The group differences in CES-D, SSQ, and RS-14 scores were investigated by using analysis of covariance (ANCOVA). Mediation analysis was conducted by using Preacher and Hayes’ bootstrap script to assess whether regular physical exercise is associated with depressive symptoms indirectly through resilience and social support.

**Results:**

The SSQ Number score (*F* = 4.82, *p* = 0.03), SSQ Satisfaction score (*F* = 6.68, *p* = 0.01), and RS-14 score (*F* = 6.01, *p* = 0.01) were significantly higher in the group with regular physical exercise (*n* = 83) than in the group without regular physical exercise (*n* = 632) after adjusting for age, education, marital status, and job status. The difference in CES-D score was not significant (*F* = 2.90, *p* = 0.09). Bootstrapping revealed significant negative indirect associations between physical exercise and CES-D score through the SSQ Number score (bias-corrected and accelerated confidence interval (BCACI) = −0.61 to −0.035; 95 % confidence interval (CI)), SSQ Satisfaction score (BCACI = −0.92 to −0.18; 95 % CI), and RS-14 score (BCACI = −1.89 to −0.094; 95 % CI).

**Conclusion:**

Although we did not find a significant direct association between exercise and depressive symptoms, exercise may be indirectly associated with depressive symptoms through social support and resilience. Further investigation is warranted.

## Background

Depressive symptoms are common in the workplace and can result in outcomes such as suicide, impaired job performance [1], long absences due to sickness [2], and the need to pay disability pensions [3]. Depressive symptoms therefore represent a substantial economic burden to society [4, 5]. Preventing the development of depressive symptoms in the workplace is therefore of great importance for both employees and employers, as well as for society as a whole. Depressive symptoms in the workplace have been associated with psychosocial factors, such as poor social support and job strain, defined as high demands and low decision latitude in the workplace [6].

Accumulated evidence has shown that moderate-intensity regular physical exercise has beneficial effects on depressive symptoms, as well as diseases such as type 2 diabetes and coronary heart disease. A meta-analysis revealed that exercise has moderate beneficial effects on depressive disorders [7], and several studies have suggested that exercise can reduce the risk of depressive symptoms in the workplace [8, 9]. Guidelines for the treatment of depressive disorders developed by the Japanese Society of Mood Disorders recommend exercise three or more times per week for mild depressive disorders [10], although the precise dose of physical exercise needed to treat depression remains elusive.

Both biological factors and psychosocial factors have been proposed as possible mechanisms for the beneficial effect of regular physical exercise on depression. Social support is an important preventive factor for depressive symptoms [11–13]. Physical exercise is often undertaken in a social environment, leading to the ‘social interaction’ hypothesis [14]. For example, contact with the person supervising the exercise in interventional trials of exercise may have provided social support, resulting in improvement of depressive symptoms [15–17]. As discussed in systematic reviews of the effects of physical exercise interventions on depressive symptoms, a number of studies did not control for variables such as social support, although participants were required to exercise under supervision or in group situations [7, 14, 16]. Thus, physical exercise may prevent depression by promoting social support.

Resilience, which is defined as a dynamic process and the ability to adapt to challenging life conditions [18–20], is key to adapting to the daily psychological burden in the workplace and to preventing the development of depressive symptoms. Resilience has been negatively associated with depressive symptoms, and positively associated with emotional regulation [21, 22]. Compared with persons with low resilience scores, persons with high resilience scores were reported to have more positive emotions even in stressful situations [22] and to have more emotional flexibility in response to a rapidly changing stressful psychological task [23]. Resilience is also associated with quick recovery from cardiovascular arousal [22]. Exercise has been shown to have effects similar to those of resilience. It is well recognized that physical exercise has a beneficial effect on positive mood [24]. Childs and de Wit demonstrated that those who reported exercising at least once per week also reported a lesser decline in positive affect after an emotional stress task than those who did not report physical exercise [25]. A meta-analysis demonstrated a positive effect of acute aerobic exercise on stress-related blood pressure responses [26]. Furthermore, exercise increases brain-derived neurotrophic factor, which protects neurons in regions of the brain such as the striatum and hippocampus in stressful situations [27, 28]. Zschucke et al. demonstrated that physical exercise activated the hippocampus, inactivated the prefrontal cortex, and reduced the cortisol response to an emotional task. Physical exercise might thus enhance resilience by regulating the hypothalamic-pituitary-adrenal axis to buffer the effect of daily stress [29]. Physical exercise may therefore prevent depression by promoting resilience. To the best our knowledge, however, no studies have investigated the association of regular physical exercise and resilience by using a validated resilience scale.

The aim of this study was to investigate differences in depressive symptoms, social support, and resilience between a group of Japanese company workers who engaged in regular physical exercise and a group of workers who did not and to determine whether regular physical exercise has an indirect association with depressive symptoms through social support and resilience.

## Methods

### Participants

We conducted a research in 6 workplaces in Kanto area of a company which agreed to cooperate. We instructed occupational health staffs in 6 workplaces of a company, and they explained the details of research to the company workers in face-to-face interviews. Participants were provided with a written explanation of the research, a consent form, and the self-report questionnaires by the company’s occupational health staff. Workers who agreed to participate in this study provided consent by returning the consent form and questionnaires by postal mail. This study was conducted by using a database which was collected in a previous study [20, 30]. Of the 15,071 workers at six separate worksites of a large company located in an urban area of Japan, 2159 workers (13.4 %) were approached. Among them, 741 (34.3 %) agreed to participate in the study. We excluded 26 participants with missing responses to items related to the subscales used, leaving 715 participants for analysis in this study. The workers who did not participate did not differ significantly from the participants in terms of age or sex.

### Measures

Demographic information on sex, marital status, educational attainment, and job status were collected by self-report.

#### Assessment of depressive symptoms

The Center for Epidemiologic Studies Depression (CES-D) questionnaire was administered to assess depressive symptoms. CES-D is a self-report questionnaire consisting of 20 items, and the scores are summed to yield a total score between 0 and 60, with a higher score indicating more severe depressive symptoms. This scale is one of the most widely used scales to assess depressive symptoms in the past week [31]. The reliability and validity of the Japanese version have been verified [32].

#### Assessment of social support

The short version of the Social Support Questionnaire (SSQ) was administered to assess social support. The short version of SSQ consists of six items with 12 questions [33]. Each item has two parts. The first part assesses the number of others to whom the individual feels he or she can turn in times of need in various situations. The second part measures the individual’s degree of satisfaction with the perceived support available in that particular situation. Responses are rated on a 6-point Likert scale (1 = “very dissatisfied”; 6 = “very satisfied,”). Two scores are obtained: the SSQ Number score for the perceived number of social supports, and the SSQ Satisfaction score for satisfaction with the social support that is available. The scores for each participant were calculated by averaging the scores of all items. Sarason et al. developed the SSQ as a reliable, valid, and convenient index of social support [34]. The Japanese version of the SSQ has been verified to be reliable and valid [35].

#### Assessment of resilience

The 14-item Resilience Scale (RS-14) was administered to assess resilience. The RS-14 is an abbreviated version of the Resilience Scale (RS), which is a self-report questionnaire consisting of 25 items that measure the degree of individual resilience [18]. Each item is rated on a 7-point Likert scale (total score range, 14–98), with a higher score indicating more resilience [18]. The RS was developed through a qualitative study of people who had experienced a recent loss (e.g., of a spouse, health, or employment) and had adapted successfully [18, 36–40]. The RS scale was recommended as an excellent and widely used scale to assess psychological resilience in a review by Ahern [41]. The RS-14 strongly correlates with the RS. The reliability and validity of the Japanese version have been verified [42].

#### Assessment of frequency of physical exercise

To evaluate physical exercise habits, we extracted a single item from the Japanese version of the Health-Promoting Lifestyle Profile [43]. Physical exercise was assessed with a frequency question: “The next question is about your physical exercise habits. In the last six months, how often did you do relatively hard exercise for more than 20 min, such as jogging or running, cycling, aerobics, and stepping exercise?.” Four response options were given for each question: 1) never, 2) 1–3 times a month, 3) 1–2 times a week, and 4) 3 or more times per week.

### Statistical analysis

All of the analyses were performed using SPSS, version 23 (SPSS Inc., Chicago). Alpha levels were all set at *p* < 0.05. We divided the participants into two groups based on their frequency of relatively hard exercise: those exercising more than 20 min, three or more times per week, were defined as the regular physical exercise group, and all others were defined as the group without regular exercise. We also dichotomized the participants by demographic data, as follows: marital status into whether married or not, educational attainment into whether graduated from college or university or not, and job status into whether in a management position or not. Age was compared between the two groups with Student’s *t* test. Differences in the categorical variables of marital status, educational attainment, and job status were analyzed with chi-square tests or Fisher’s exact test. The group differences in CES-D score, SSQ Number score, SSQ Satisfaction score, and RS-14 score were compared between the groups with and without regular physical exercise after adjusting for age, sex, marital status, educational attainment, and job status by using analysis of covariance (ANCOVA).

**Fig. 1:**
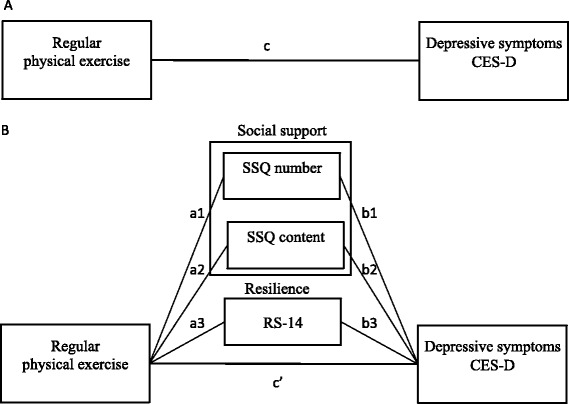
Models of associations between exercise and depressive symptoms. **a** Illustration of a direct association between regular physical exercise and depressive symptoms. Path *c* represents the total effect of regular physical exercise on the total score of the Center for Epidemiologic Studies Depression (CES-D) scale. **b** Illustration of an indirect association between regular physical exercise and depressive symptoms (CES-D) mediated by resilience (14-item Resilience Scale, RS-14) and social support (Social Support Questionnaire, SSQ). The paths *a*_*n*_ represent the association between regular physical exercise and each mediator. The paths *b*_*n*_ represent the association between each mediator and depressive symptoms (CES-D). Path *c′* is the association between the regular physical exercise and depressive symptoms (CES-D), without mediators

Additionally, to investigate indirect associations between regular physical exercise and depressive symptoms through social support and resilience, we conducted a mediation analysis using the statistical analysis framework defined by Baron and Kenny [44], as follows. First, a regression analysis was conducted to evaluate the *c* path (Fig. 1), in which CES-D score was the dependent variable and regular physical exercise was the independent variable. Second, regression analysis was conducted to evaluate the *a*_*n*_ paths; each mediator variable (*n* = 1: SSQ Number score; *n* = 2: SSQ Satisfaction score; *n* = 3: RS-14 score) was entered as a dependent variable, and regular physical exercise was the independent variable. Third, regression analysis was conducted to evaluate the *b*_*n*_ paths and *c′* path*,* with CES-D score as the dependent variable and each mediator variable as an independent variable. Next, the sizes of the indirect associations between regular physical exercise and the CES-D score through SSQ Number score (*a*_*1*_ × *b*_*1*_), SSQ Satisfaction score (*a*_*2*_ × *b*_*2*_), and RS-14 score (*a*_*3*_ × *b*_*3*_) were estimated, using a bias-corrected bootstrapping method [45] with 5000 replications, and bootstrap 95 % confidence intervals (CIs) were obtained. The mediation model and any indirect associations were assessed by using Preacher and Hayes’ bootstrap script for SPSS [45], which can handle nonparametric data. The CES-D score was the dependent variable; regular physical exercise was entered as the independent variable; the RS-14, SSQ Number, and SSQ Satisfaction scores were entered as mediator variables; and age, sex, marital status, educational attainment, and job status were entered as control variables. When the bootstrap 95 % CI did not include zero, the indirect association was taken to be significant, equivalent to testing for significance at the 0.05 level.

## Results

### Demographics

All 715 participants were Japanese. Other demographic characteristics, and mean scores in the CES-D, SSQ, and RS-14 instruments, are shown in Table 1. In a univariate analysis of background variables and regular physical exercise, only low educational attainment was significantly associated with regularly engaging in physical exercise (*p* < 0.01; Table 2).

**Table 1:** Demographic characteristics

		Number	Percent
Regular physical exercise	Yes	83	(11.3)
Sex	Male	596	(83.4)
Marital status	Married	466	(65.2)
Educational attainment	Graduated from university or college	590	(82.5)
Job status	Management position	67	(9.4)
		Mean	SD
Age		39.9	9.4
CES-D		10.3	7.6
SSQ(Number)		3.9	2.4
SSQ(Satisfaction)		4.7	1.0
RS-14		64.0	11.2

*CES-D* Center for Epidemiologic Studies Depression, *RS-14* 14-item Resilience Scale, *SSQ* social support questionnaire, *SD* standard deviation

**Table 2:** Univariate analysis of the relationship between demographic variables and regular physical exercise

		Regular physical exercise				
		Yes		No		
		*n* = 83(11.8 %)		*n* = 632(83.2 %)		
		Mean	(SD)	Mean	(SD)	*p*
Age		39.9	(9.3)	39.9	(9.8)	0.96
		n	(%)	n	(%)	p
Sex	Male	75	(12.6)	521	(87.4)	0.06
	Female	8	(6.7)	111	(93.3)	
Married	yes	52	(62.7)	414	(65.5)	0.63
	no	31	(12.4)	218	(87.6)	
Graduated from university or college	yes	58	(9.8)	532	(90.2)	<0.01*
	no	25	(20.0)	100	(80.0)	
Management position	yes	6	(9.0)	61	(91.0)	0.69
	no	77	(11.9)	571	(88.1)	

*p < 0.05. Regular physical exercise: yes, frequency of relatively hard exercise, more than 20 min, three or more times per week; no, lower frequency or intensity of exercise

### Regular physical exercise and depressive symptoms, social support, and resilience

There was no significant difference in CES-D score between the group with regular physical exercise and the group without regular physical exercise (*F* = 2.90, *p* = 0.09; Table 3). The group with regular physical exercise had significantly higher SSQ Number score (*F* = 4.82, *p* = 0.03), SSQ Satisfaction score (*F* = 6.68, *p* = 0.01), and RS-14 score (*F* = 6.01, *p* = 0.01).

**Table 3:** Depressive symptoms, social support, and resilience in the groups with and without regular physical exercise

	Regular physical exercise					
	Yes		No			
	*n* = 83(11.9 %)		*n* = 632(83.2 %)			
	Mean	(SD)	Mean	(SD)	F^a^	p^a^
CES-D	9.2	(7.4)	10.4	(7.6)	2.90	0.09
SSQ(Number)	4.4	(2.5)	3.8	(2.3)	4.82	0.03*
SSQ(Satisfaction)	5.0	(0.8)	4.7	(1.0)	6.68	0.01*
RS-14	66.5	(12.8)	63.7	(10.1)	6.08	0.01*

*CES-D* center for epidemiologic studies depression, *RS-14* 14-item Resilience Scale, *SSQ* social support questionnaire, *SD* standard deviation

^a^Adjusted for age, sex, marital status, educational attainment, and job status

* *p* < 0.05

### Indirect association between regular physical exercise and depressive symptoms through social support and resilience

The results of the regression analysis using Preacher and Hayes’ bootstrap script are as follow. There was no significant association between regular physical exercise and CES-D score (*c* path: B = −1.51, SE = 0.89, *p* = 0.09). After controlling for all mediator variables, SSQ Number score, SSQ Satisfaction score, and RS-14 score, no significant association remained between regular physical exercise and CES-D score (c′ path: B = −0.17, SE = 0.72, *p* = 0.81). Regular physical exercise was significantly associated with SSQ Number score (*a*_*1*_ path: B = 0.6, SE = 0.28, *p* = 0.03), SSQ Satisfaction score (*a*_*2*_ path: B = 0.29, SE = 0.11, *p* < 0.01), and RS-14 score (*a*_*3*_ path: B = 3.20, SE = 1.30, *p* = 0.01). CES-D score was significantly associated with SSQ Number score (*b*_*1*_ path: B = 0.6, SE = 0.28, *p* = 0.03), SSQ Satisfaction score (*b*_*2*_ path: B = −1.63, SE = 0.26, *p* < 0.01), and RS-14 score (*b*_*3*_ path: B = −0.30, SE = 0.02, *p* < 0.01).

The bootstrapping results revealed that there was a significant negative indirect association between physical exercise and CES-D score through the SSQ Number score (bias-corrected and accelerated confidence interval (BCACI)= −0.61 to −0.0350; 95 % confidence interval (CI)), SSQ Satisfaction score (BCACI = −0.92 to −0.18; 95 % CI), and RS-14 score (BCACI = −1.89 to −0.094; 95 % CI).

## Discussion

We investigated the association between physical exercise and depressive symptoms, social support, and resilience in Japanese workers. The participants in the current study were mainly men who were highly educated and worked for a large Japanese company that provides good job security and a relatively good balance of effort and reward. Only 11.6 % of participants indicated that they engage in regular physical exercise, which we defined as at least 20 min, three or more times per week, as recommended by the guidelines for treating depressive disorders from the Japanese Society of Mood Disorders.

CES-D scores were numerically lower in participants who engaged in regular physical exercise, but this did not reach statistical significance in the ANCOVA analysis. This result does not seem to support previous findings, which demonstrated a benefit of physical exercise on depressive symptoms [7, 46, 47]. This might be because our participants did not have depressive symptoms severe enough to prevent them from performing the routine duties of their company jobs. Accumulated evidence supports depression as a continuum of disorders, with severity being the only difference between major depression and minor depression [48]. Consistent with our results, a previous randomized, controlled, intervention study of a workplace physical exercise program for white-collar employees with minimal symptoms of depression did not show a statistically significant improvement compared with a control group [9]. Thus, exercise might have more limited effects in individuals with mild depressive symptoms.

Another possible explanation for our results is the dose of physical exercise. There have been several studies showing a U-shaped association between physical exercise and depressive symptoms [49–51]. The risk of depressive symptoms was found to gradually decrease from no exercise to a high dose of leisure-time exercise (16.5 to <25 metabolic equivalent [MET] hours per week), and then to slightly increase again at a very high dose (above 25.5 MET hours per week) in a cohort study of Japanese company workers [49]. A U-shaped association was also found between vigorous-intensity exercise and depressive symptoms in a cohort study of American Black women, with the greatest risk reduction (18 %) occurring at 3–4 h per week of vigorous exercise [50]. The dose of physical exercise in the present study therefore may not be enough to alleviate depressive symptoms, or very high doses of exercise in some participants might have attenuated the benefits of exercise on depressive symptoms.

However, the result of the current study suggest that regular exercise might have a benefit on depressive symptoms in the workplace through social support and resilience. The ANCOVA analysis indicated that participants engaging in regular exercise had significantly higher social support and resilience compared with those who did not engage in regular physical exercise. Furthermore, in the mediation analysis, the bootstrap result showed a statistically significant indirect association between depressive symptoms and physical exercise through resilience and social support. Although our results did not meet the statistical framework in which Baron and Kenny [44] defined mediation as occurring if the *a*_*n*_*, b*_*n*_*, c* paths are significant and the *c*′ path is not significant, because the *c* path was not significant in our analysis, some authors have proposed that a significant total effect is not necessary to show mediation if the indirect effect is significant [52, 53]. Thus the findings of this study were not inconsistent with the hypothesis that regular physical exercise attenuates depressive symptoms in part by promoting social support and resilience, but further investigation is warranted.

Chou reported a beneficial effect of Tai Chi, a traditional Chinese exercise, on depressive symptoms, but found that the effect disappeared when changes in social support were controlled for, indicating that social support might be partly responsible for the effect of the exercise on depressive symptoms. Many kinds of physical exercise need a supervisor or instructor, some require a partner, and some are played in groups or teams. The improvements in mental health following physical exercise are at least partly related to the mutual support and social relationships that are provided when participating in physical exercise with others [54].

Although there are several lines of evidence linking resilience to regular physical exercise, to the best our knowledge, this is the first study to investigate the association between regular physical exercise and resilience by using a validated resilience scale. In this study, only 11.6 % of participants engaged in regular physical exercise. It might not be easy for a busy company worker to get into the habit of regular physical exercise. Substantial drop-out rates have been reported in studies of physical exercise interventions [7], and sustaining physical exercise as a fitness habit for the long term is difficult, although it is important for preventing depressive symptoms [55]. Developing the habit of physical exercise itself might reinforce self-esteem because it is a difficult accomplishment; this is one proposed mechanism for the effect of physical exercise on depressive symptoms [56]. Interventional studies for the prevention of depression might also produce resilience even in the absence of a significant change in depressive symptoms themselves. In fact, several approaches that increase resilience, such as well-being therapy, are used to treat depression, not by attenuating and preventing negative symptoms but by promoting positive emotions in order to increase psychological well-being [57–60].

Our study had several limitations. First, due to the cross-sectional nature of the study design, causal relationships between the factors could not be determined. It is also possible that social support and resilience attenuated the depressive symptoms through regular physical exercise, rather than the effects of exercise being mediated by social support and resilience. However, the findings of the current study do not seem to support such mediation models, because the association between regular physical exercise and depression (b path) was weaker than the association between resilience and depressive symptoms and did not reach statistical significance. There are also possible mutual or bidirectional associations among physical activity, social support, and resilience. These associations might be helpful for developing the habit of physical exercise. Second, a response rate was not satisfactory; we could not exclude the risk of the bias. Those who were depressed and sedentary might be reluctant to participate in the research compared with those were not depressed and active such as with regular physical exercise. These biases may attenuate the association among regular exercise, resilience, and depression. No statistically significant difference in CES-D score between the group with regular physical exercise and the group without regular physical exercise in current study might be due to this low response rate. Third, the participants were mainly men, they were highly educated, and they worked for a large Japanese company that provides good job security and a relatively good balance of effort and reward. The company worker from a single large company may be a vulnerable subject and have a potential about deviated report. These characteristics leave open the possibility that the participants are not representative of workers more generally. Further studies should be conducted in a community level or a multi-company level. Forth, information on the frequency of exercise was self-reported, and nondifferential misclassification may be inevitable and could attenuate the observed associations. Finally, residual confounding by uncontrolled or unmeasured factors may have distorted genuine associations.

## Conclusion

We assessed the association between regular physical exercise, which is recommended by guidelines for maintaining health, and depressive symptoms in Japanese company workers, taking into account social support and resilience. The results suggest that regular physical exercise might not affect depressive symptoms directly, but might attenuate depressive symptoms indirectly through social support and resilience. In conclusion, the findings of the current study are not inconsistent with regular exercise providing a benefit for reducing depression through social support and resilience, but further investigation is warranted.

## Abbreviations

ANCOVA, analysis of covariance; BCACI, bias-corrected and accelerated confidence interval; CES-D, The Center for Epidemiologic Studies Depression; CI, confidence interval; RS-14, 14-item resilience scale; SSQ, Short version of Social Support Questionnaire
